# The Machine Learning Model for Distinguishing Pathological Subtypes of Non-Small Cell Lung Cancer

**DOI:** 10.3389/fonc.2022.875761

**Published:** 2022-05-26

**Authors:** Hongyue Zhao, Yexin Su, Mengjiao Wang, Zhehao Lyu, Peng Xu, Yuying Jiao, Linhan Zhang, Wei Han, Lin Tian, Peng Fu

**Affiliations:** ^1^ Department of Nuclear Medicine, The First Affiliated Hospital of Harbin Medical University, Harbin, China; ^2^ Department of Magnetic Resonance, The First Affiliated Hospital of Harbin Medical University, Harbin, China; ^3^ Department of Pathology, The First Affiliated Hospital of Harbin Medical University, Harbin, China

**Keywords:** [^18^F]F-FDG PET/CT, radiomics, lung adenocarcinoma, lung squamous cell carcinoma, machine learning

## Abstract

**Purpose:**

Machine learning models were developed and validated to identify lung adenocarcinoma (LUAD) and lung squamous cell carcinoma (LUSC) using clinical factors, laboratory metrics, and 2-deoxy-2[^18^F]fluoro-D-glucose ([^18^F]F-FDG) positron emission tomography (PET)/computed tomography (CT) radiomic features.

**Methods:**

One hundred and twenty non-small cell lung cancer (NSCLC) patients (62 LUAD and 58 LUSC) were analyzed retrospectively and randomized into a training group (n = 85) and validation group (n = 35). A total of 99 feature parameters—four clinical factors, four laboratory indicators, and 91 [^18^F]F-FDG PET/CT radiomic features—were used for data analysis and model construction. The Boruta algorithm was used to screen the features. The retained minimum optimal feature subset was input into ten machine learning to construct a classifier for distinguishing between LUAD and LUSC. Univariate and multivariate analyses were used to identify the independent risk factors of the NSCLC subtype and constructed the Clinical model. Finally, the area under the receiver operating characteristic curve (AUC) values, sensitivity, specificity, and accuracy (ACC) was used to validate the machine learning model with the best performance effect and Clinical model in the validation group, and the DeLong test was used to compare the model performance.

**Results:**

Boruta algorithm selected the optimal subset consisting of 13 features, including two clinical features, two laboratory indicators, and nine PEF/CT radiomic features. The Random Forest (RF) model and Support Vector Machine (SVM) model in the training group showed the best performance. Gender (P=0.018) and smoking status (P=0.011) construct the Clinical model. In the validation group, the SVM model (AUC: 0.876, ACC: 0.800) and RF model (AUC: 0.863, ACC: 0.800) performed well, while Clinical model (AUC:0.712, ACC: 0.686) performed moderately. There was no significant difference between the RF and Clinical models, but the SVM model was significantly better than the Clinical model.

**Conclusions:**

The proposed SVM and RF models successfully identified LUAD and LUSC. The results indicate that the proposed model is an accurate and noninvasive predictive tool that can assist clinical decision-making, especially for patients who cannot have biopsies or where a biopsy fails.

## Introduction

In 2020, about 1.8 million people died of lung cancer, accounting for one-fifth of cancer-related deaths (1). Of these, 80%–85% were cases of non-small cell lung cancer (NSCLC), of which lung adenocarcinoma (LUAD, ~50%) and lung squamous cell carcinoma (LUSC, ~40%) are the most common subtypes (2, 3). Due to the different histologic and biological characteristics of LUAD and LUSC, there are significant differences in their treatment regimen, prognosis, and relapse rates (4, 5). For example, targeted drugs can significantly improve the prognosis of patients with LUAD in genetic mutations such as epidermal growth factor receptor or anaplastic lymphoma kinase. By contrast, treatment options for advanced LUSC have limited first-line and relapsed/refractory settings (6). Additionally, a concurrent chemotherapy regimen combined with pemetrexed and cisplatin/carboplatin can be adopted after NSCLC surgery (7). Scagliotti et al. found that pemetrexed significantly prolonged the overall and progression-free survival of LUAD patients but reported the opposite effects for LUSC patients (8). Accordingly, distinguishing between these two NSCLC subtypes before treatment is critical for clinical decisions. In current clinical practice, bronchoscopy and computed tomography (CT)-guided biopsies are the gold standards for recognizing lung cancer subtypes. Unfortunately, as an invasive examination, contraindications and complications of manipulation are unavoidable. Moreover, a repuncture biopsy is more difficult when less pathological tissue is obtained from the first puncture and does not meet the requirements for accurate diagnosis (9). Of note, tumor heterogeneity can also have an impact on biopsy results. It is wise to explore non-invasive tools that can assist NSCLC subtype judgment.

Although clinicians can distinguish NSCLC subtypes based on different imaging characteristics and clinical manifestations, experience-based judgment is complex as an accurate and quantitative means of measurement. Some reports testify that indicators such as 2-deoxy-2[^18^F]fluoro-D-glucose ([^18^F]F-FDG) positron emission tomography (PET)/CT radiomic features (4, 10–14) can all provide quantitative biomarkers for distinguishing NSCLC subtypes. These promising preliminary results motivated further studies to develop noninvasive detection methods to identify NSCLC subtypes for the purpose of early diagnosis and treatment. Radiomics, in particular, is called a virtual biopsy (15). This technique enables a noninvasive and comprehensive quantification of tumor phenotypes by converting medical images into diggable high-dimensional quantitative data (16). Deep mining of radiomic features using machine learning techniques proved effective in accurately distinguishing LUAD from LUSC (4, 10–14). As mentioned above, however, tumors are heterogeneous, and a single factor is often insufficient for a complete description of the overall situation of the tumor lesion. Clinical factors and laboratory indicators are critical in distinguishing LUAD from LUSC in different ways (10, 11). Ren et al. attempted to evaluate the value of clinical factors, laboratory indicators and radiomics features in identifying NSCLC subtypes. Their results showed that combining clinical factors and laboratory indicators could further improve the prediction effect of radiomic features (10). However, this is a preliminary study and the model is simple. There are many machine learning algorithms available, and only a few experiments have explored which model is more suitable for the classification task of NSCLC (11, 12). Multiple factors and multiple models can facilitate comprehensive evaluations of tumors and promote the construction of more accurate models.

As such, this study mainly wants to solve two problems. The first is to identify machine learning algorithms suitable for the classification task of identifying NSCLC. The second is to develop a precise machine learning model that combines clinical factors, laboratory indicators, and radiomic features to assist in identifying NSCLC pathological subtypes.

## Materials and Methods

### Patients

The retrospective investigation consisted of 210 lung cancer patients treated at the First Affiliated Hospital of Harbin Medical University (Harbin, China) between January 2016 and December 2020. The inclusion criteria were as follows (1): patients were pathologically diagnosed as LUAD or LUSC (2); no anti-tumor treatment was performed before performing the [^18^F]F-FDG PET/CT scan; and (3) no history of other malignancies. The exclusion criteria were as follows (1): the size of the primary tumor lesion was not enough for texture analysis (LIFEx software only calculates the texture features of lesions with ≥ 64 voxels) (2); the primary lesion or its boundaries could not be identified by the PET image (3); clinical data, including gender, age, smoking status, and family history, and/or laboratory indicators, including carcinoembryonic antigen (CEA), squamous cell carcinoma antigen (SCCA), cytokeratin 19 fragment antigen21-1 (CA211), and neuron-specific enolase (NSE), were absent.

A total of 120 NSCLC patients were included in the study: 62 patients with LUAD and 58 patients with LUSC. The patients were first divided into a training group (n = 85) and a validation group (n = 35), and the R package “createDataPartition” function with “caret” was used to divide the dataset completely randomly by positive and negative sample ratios. The proportion of positive and negative samples in the training and validation groups was roughly the same as the complete dataset. The training group data were used for model training adjustment, and validation group data were used to evaluate the generalization ability of the model. This study was approved by the ethics review board of the First Affiliated Hospital of Harbin Medical University, and the informed consent was waived because of the study’s retrospective properties.

### PET/CT Image Acquisition

All patients were required to fast for 6–8 hours, and venous blood glucose levels were controlled to less than 8.0 mmol/L. In the patient’s dorsal or elbow vein, 3.7–7.4 MBq/kg of [^18^F]F-FDG isotope (HM-12, Sumitomo Heavy Industries Ltd., Tokyo, Japan, radiochemical purity > 95%) was intravenously injected. After urinating in quiet, light-avoidance conditions (60 ± 5 min), the PET/CT images were acquired using a 16-slice Gemini GXL PET/CT scanner (Philips Medical System). A low-dose CT scan (tube voltage: 120 kV, tube current: 50 mAs, slice thickness: 5.0 mm, pitch: 1.0) was acquired for attenuation correction, and then the PET images were acquired (1.5 min per bed position, 6–7 PET bed positions). According to the agency’s standard clinical protocols, the scan range was from the head to the mid-thigh. The line of response reconstruction algorithm was used to reconstruct the image without post-reconstruction filtering after automatic random and scattering correction.

### Radiomic Feature Extraction and Evaluation

The lung cancer lesions in the PET images were analyzed slice by slice by two independent nuclear medicine physicians (Reader 1 and Reader 2) using LIFEx software (version 7.0.0, http://www.lifexsoft.org) (17), and the volume of the area of interest (VOI) was automatically delineated at a threshold of 40% of the maximum standardized uptake value (SUV). Absolute resampling was used for spatial resampling. VOI voxels were resampled to 4.0 × 4.0 × 4.0 mm, the range of the SUV was set at 0.0–20.0, and the number of grey levels was set to 64.0 bins (bin width: 0.3125). The same VOI was used for the extraction of the CT feature parameters. During the extraction of CT feature parameters, voxels within the VOI were resampled to 2.0 × 2.0 × 2.0 mm, the range of CT values was set to −1000.0–3000.0 HU, and the number of grey levels was selected to 400.0 bins (bin width: 10). Finally, 47 PET feature parameters (six conventional indices, nine first-order features, and 32 second-order features) and 45 CT feature parameters (four conventional indices, nine first-order features, and 32 second-order features) were extracted. The formulation of the feature parameters is available at http://www.lifexsoft.org.

**Figure 1:**
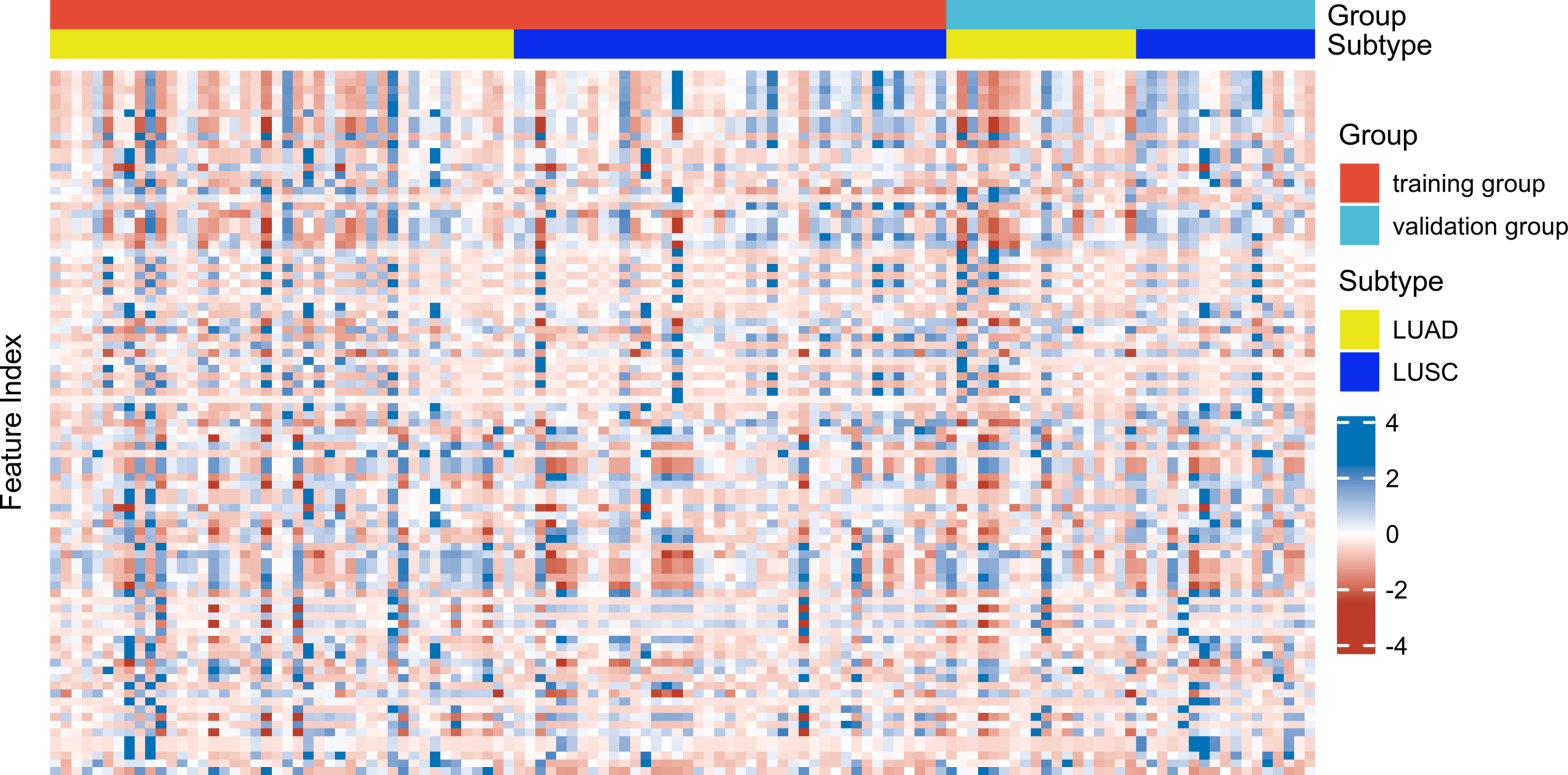
Heatmap of 91 radiomic features (in columns) distributed in the training group (n = 85) and (in rows) distributed in the validation group (n = 35).

Reader 1 completed VOI segmentation in all patients. After 14 days apart, 20 patients (10 LUAD patients and 10 LUSC patients) were randomly selected. Reader 1 and Reader 2 each segmented regions of interest for delineation and feature extraction. The two observers were blinded to each other and blinded to the histopathological diagnosis. Inter-observer and intra-observer agreement of tumor segmentation was assessed by inter-and intra-class correlation coefficients (ICCs). When the inter-observer and intra-observer ICCs were > 0.75, the feature was considered good reproducibility and was retained. The ICCs between the two observers reached 0.982 ± 0.036, ranging from 0.741–1.000. The ICCs within the same observer reached 0.986 ± 0.036, with a range from 0.725–1.000. Only one feature (grey-level zone-length matrix (GLZLM)_Long-zone low grey-level emphasis (LZLGE)^PET^) was deleted due to poor reproducibility (intra-observer ICCs = 0.725; inter-observer ICCs = 0.741). Thus, 91 PET/CT radiomic features were used for the subsequent experiments. A heatmap of the radiomic features in the training and validation groups is shown in **Figure 1**.

### Feature Selection

In the training group cohort, 99 characteristic parameters were screened: four clinical characteristics (gender, age, smoking status, and family history), four laboratory indicators (CEA, SCCA, NSE, and CA211), and 91 PET/CT radiomic features. All features but three classification features (gender, smoking status, and family history) were processed by z-score standardization. This increased the classification accuracy, as the more extensive numerical range had more minor effects on the prediction. Then, the Boruta algorithm was performed to further feature screening (18), which belongs to a wrapper algorithm. This algorithm adds randomness to a given dataset by creating shadow features. It then iterates to check if a natural feature is more important than the best shadow feature and continually removes features it deems very unimportant. Finally, the algorithm outputs a minimum and optimal subset of features.

### Model Building and Validation

The filtered features were brought into ten machine learning classifiers, including Logistic Regression (LR), Linear Discriminant Analysis (LDA), Naive Bayes (NB), K-Nearest Neighbor (KNN), Support Vector Machine (SVM) with radial basis function kernel, Decision Tree (DT), Random Forest (RF), eXtreme Gradient Boosting (XGBoost), AdaBoost and Artificial Neural Network (ANN). The best-performing model was selected by comparing the area under the receiver operating characteristic curve (AUC) and accuracy (ACC) values. The control parameters of the best model were further optimized by grid search and ten-fold cross-validation.

Then, the Clinical model was constructed. First, a univariate analysis of clinical factors and laboratory indicators was performed to obtain statistically different distribution variables between the LUAD and LUSC groups. The variance inflation factor (VIF) of these different distribution variables was calculated to ensure no collinearity between variables. The differential variables were input into forwarding stepwise regression for obtaining the independent risk factors distinguishing LUAD and LUSC. The regression equation was listed to construct the Clinical model.

Subsequently, the model’s effect was validated in the validation group cohort (n = 35) using AUC, sensitivity (SEN), specificity (SPE), and ACC. The DeLong test was used to compare the performance of the models.

### Statistical Analysis

Data analyses were performed using SPSS software version 25.0 (SPSS, Chicago, IL, USA). Continuous variable data are presented as the mean ± standard deviation, and categorical variables are offered as rates or percentages. Differences in the clinical data distribution in the training and validation groups were compared using the t-test or chi-squared test. Univariate analysis was performed by t-test/Mann-Whitney U test and chi-squared test. Drawing and machine learning models were performed using R language (version 3.6.3, http://www.r-project.org) packages: “irr”, “caret”, “Boruta”, “e1071”, “glm”, “stepLDA”, “nb”, “knn”, “svmRadial”, “rpart”, “ranger”, “xgbTree”, “ada”, “nnet”, “ggplot2”, “pROC”, “reportROC”, etc. A two-tailed P value < 0.05 was considered statistically significant.

## Results

### 1. Demographic and Clinical Characteristics of the Patients

A total of 120 NSCLC patient samples were collected in this study: 62 patients with LUAD (51.7%), the mean age of 60.92 ± 10.38 years, 82 men (66.7%); 55 patients with smoking history (46.7%), and 11 patients with family history (8.9%). Then, 70% of the total sample was chosen using hierarchical random sampling to train the model, and the remaining 30% was used as a validation group to evaluate the model performance. The training group contained 85 patients (44 patients with LUAD, 58 men and 27 women, with a mean age of 61.33 ± 10.79 years), and the validation group included 35 patients (18 patients with LUAD, 24 men and 11 women, with a mean age of 59.94 ± 9.39 years). There was no statistically significant difference (P > 0.05) between the training and validation groups. Details of the demographic and clinical characteristics of the training and validation cohorts are presented in **Table 1**.

**Table 1:** Demographic differences in the training and validation cohorts.

Characteristic	Training group(n=85)	Validation group(n=35)	P value
Subtype (%)			0.973^a^
LUAD	44 (51.8)	18 (51.4)	
LUSC	41(48.2)	17 (48.6)	
Gender (%)			0.971^a^
Men	58 (68.2)	24 (68.6)	
Women	27 (31.8)	11 (31.4)	
Age (years, mean±SD)	61.33±10.79	59.94±9.39	0.508^b^
Smoking status (%)			
yes	39 (45.9)	16 (45.7)	0.987^a^
no	46 (54.1)	19 (54.3)	
Family history			
yes	22 (25.9)	6 (17.1)	0.304^a^
no	63 (74.1)	29 (82.9)	

There is no statistically significant difference (P > 0.05) between the training and validation groups.

a Chi-square test.

b Student’s t-test.

Lung adenocarcinoma, LUAD; Lung squamous cell carcinoma, LUSC; Standard deviation (SD).

### 2. Feature Selection Results

**Figure 2:**
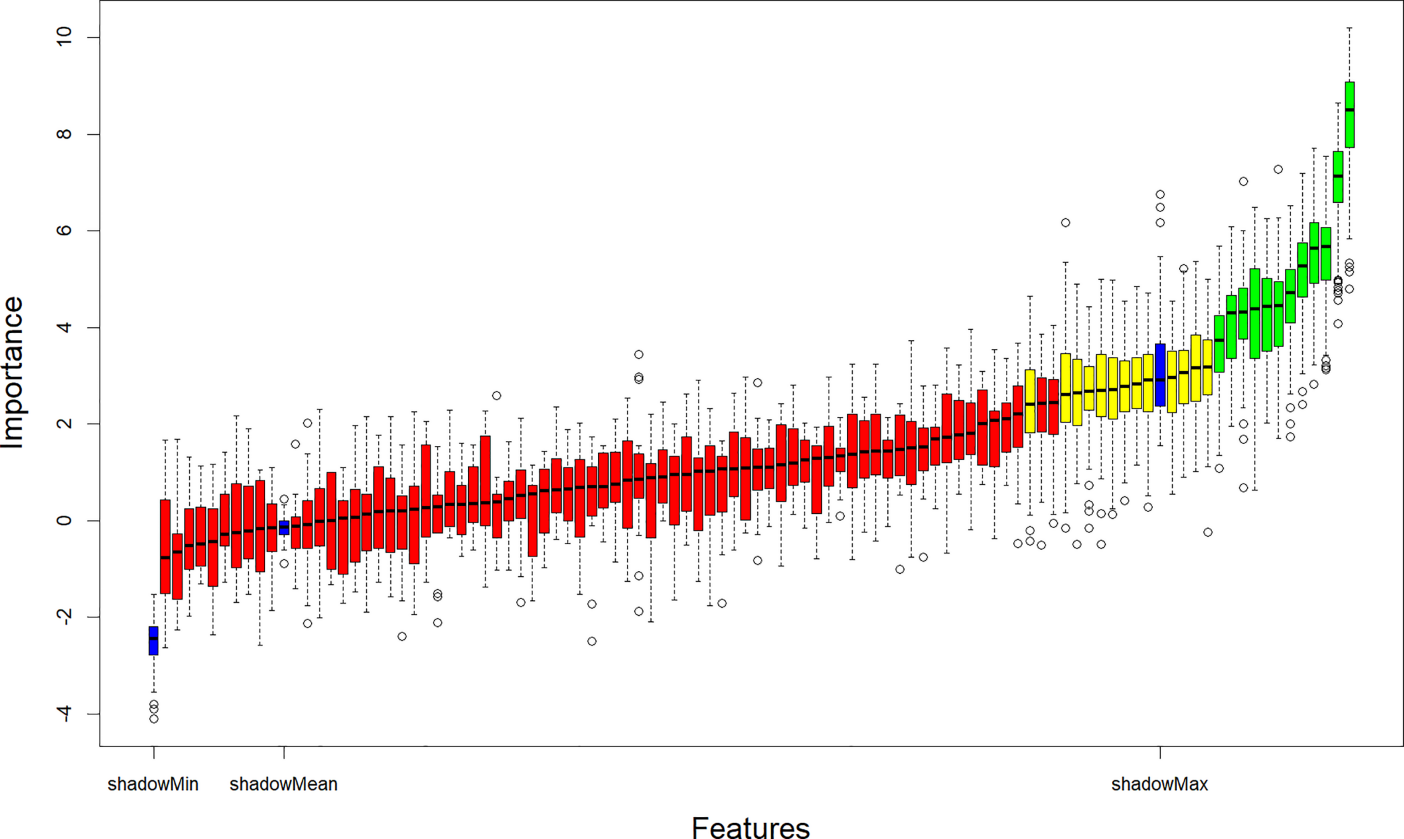
Relevant features (highlighted in green) to the NSCLC subtypes selected with the Boruta algorithm. NSCLC, Non-small cell lung cancer.

The Boruta algorithm was used to filter 99 features in the training cohort. Boruta algorithm determines the threshold by creating shadow features. It divided the input features into 12 confirmed important features, 13 tentative features, and 74 confirmed unimportant features. The iterative results of the feature screening process are shown in **Figure 2**. Eventually, an optimal subset of 13 essential features, including two clinical characteristics (gender and smoking status), two laboratory indicators (CEA and SCCA), and nine radiomic features (Grey-level run-length matrix (GLRLM)_Short-run high grey-level emphasis (SRHGE)^PET^, Neighbourhood grey-level different matrix (NGLDM)_Busyness^PET^, GLZLM_Short-zone low grey-level emphasis (SZLGE)^PET^, CONVENTIONAL_HUmean^CT^, DISCRETIZED_AUC_CSH^CT^, Grey-level co-occurrence matrix (GLCM)_Correlation^CT^, Grey-level run-length matrix (GLRLM)_ High grey-level run emphasis (HGRE)^CT^, GLZLM_ Grey-level non-uniformity for the zone (GLNU)^CT^, GLZLM_ Zone length non-uniformity (ZLNU)^CT^) were output to construct the machine learning models.

### 3. Development of the Machine Learning Model

**Figure 3:**
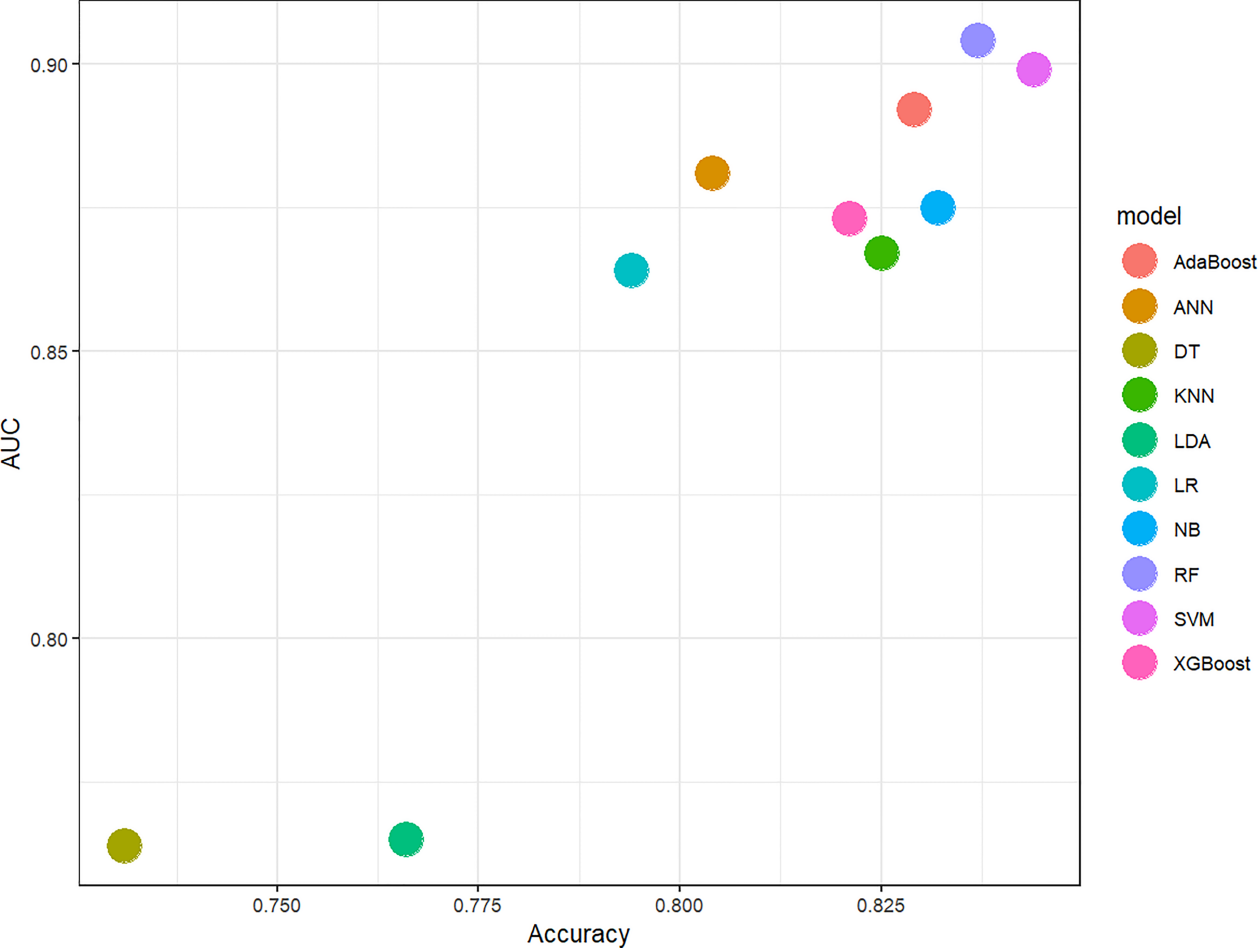
Scatter diagram of machine learning classifiers prediction performance. The horizontal axis represents ACC, the vertical axis represents AUC. AUC, The area under the receiver operating characteristic curve; ACC, Accuracy; LR, Logistic Regression; LDA, Linear Discriminant Analysis; NB, Naive Bayes; KNN, K-Nearest Neighbor; SVM, Support Vector Machine; DT, Decision Tree; RF, Random Forest; XGBoost, eXtreme Gradient Boosting; ANN, Artificial Neural Network.

**Figure 4:**
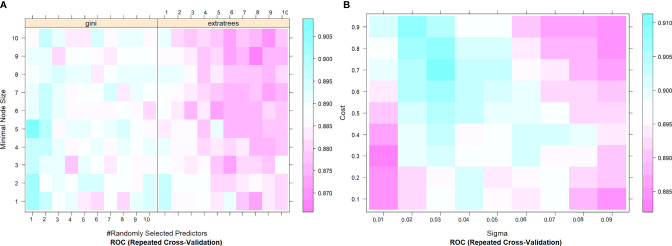
The tuning parameter grid of SVM **(A)** and RF **(B)** machine learning classifier. The gallery can be used to examine the relationship between the estimates of performance and the tuning parameters. The closer the square color is to blue, the higher the AUC is, while the closer the square color is to red, the lower the AUC value is. AUC, The area under the receiver operating characteristic curve; SVM, Support Vector Machine; RF, Random Forest.

In this study, 10 machine learning models all showed good predictive performance, and the evaluation indexes of model performance are shown in **Figure 3**. RF (AUC: 0.904, ACC: 0.837) and SVM (AUC: 0.899, ACC: 0.844) models showed the best prediction performance. DT (AUC: 0.764, ACC:0.731) and LDA (AUC: 0.765, ACC:0.766) models performed the worst. The rest of the LR (AUC: 0.864, ACC:0.794), NB (AUC:0.875, ACC: 0.832), KNN (AUC: 0.867, ACC: 0.825), XGBoost (AUC: 0.873, ACC: 0.821), AdaBoost (AUC: 0.892, ACC: 0.892) and ANN (AUC:0.881, ACC: 0.804) classification models have medium performance. Considering the two indicators comprehensively, the parameters of RF and SVM models were further optimized and adjusted by using ten-fold cross-validation and grid search techniques based on AUC value. The parameter tuning results are shown in **Figures 4A, B**. The AUC value of RF model (mtry = 1, splitrule = “gini”, min.node.size = 5) after parameter tuning was 0.906, and the AUC value of SVM model (sigma = 0.03, C = 0.7) after parameter tuning was 0.910.

### 4. Clinical Model

**Figure 5:**
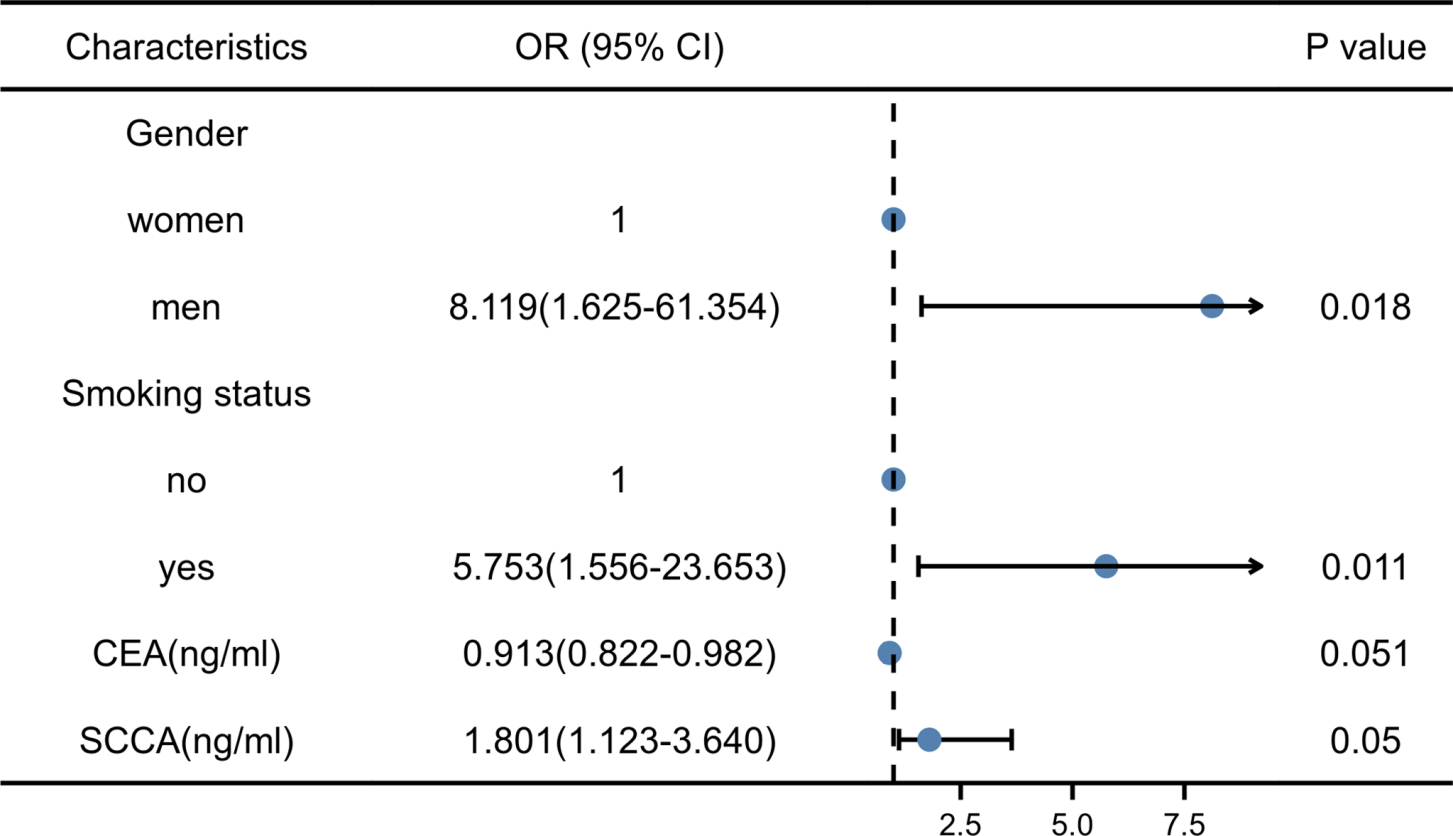
Results of stepwise regression of clinical factors and laboratory indicators. CEA, Carcinoembryonic antigen; SCCA, squamous cell carcinoma antigen; OR, Odd ratio.

Thirty-eight men with LUSC (92.7%) and three women with LUSC (7.3%). Thirty patients with a smoking history had LUSC (73.2%), and 11 patients without a smoking history had LUSC (26.8%). Pearson’s Chi-square test showed that the risk of LUSC was significantly higher in men with smoking history than in women without smoking history (all P < 0.001). The median of CEA was 3.040ng/ml in LUSC patients and 7.370 ng/ml in LUAD patients. The median of SCCA was 1.300ng/ml in LUSC patients and 0.900ng/ml in LUAD patients. Mann-Whitney U test showed that CEA and SCCA were significantly different in LUSC and LUAD groups (P =0.001 and 0.004, respectively). There were no significant differences in age (P=0.788), family history (P=0.424), NSE(P=0.327), and CA211 (P=0.342) between LUSC and LUAD groups. The VIF of gender (VIF= 1.079), smoking status (VIF=1.111), CEA (VIF=1.159), and SCCA (VIF=1.146) were all less than 10. Therefore, the differential variables were input into forwarding stepwise regression for evaluating the effects on NSCLC subtypes. The risk of LUSC was 8.119 times higher in men than in women and 5.753 times higher in patients with a smoking history than in those without a smoking history. In addition, the risk of LUSC increased by 0.913-fold for each 1-unit increase in CEA and 1.801-fold for each 1-unit increase in SCCA. Among the four variables, gender (P=0.018) and smoking history (P=0.011) could be used as independent risk factors to distinguish NSCLC subtypes, and the results of stepwise regression are shown in **Figure 5**. The Clinical model (y=2.182*Gender+1.885*Smoking status-2.560) (Gender: women=0, men=1; Smoking status: no=0, yes=1) included gender and smoking history.

### 5. Predictive Performance of the Model

**Figure 6:**
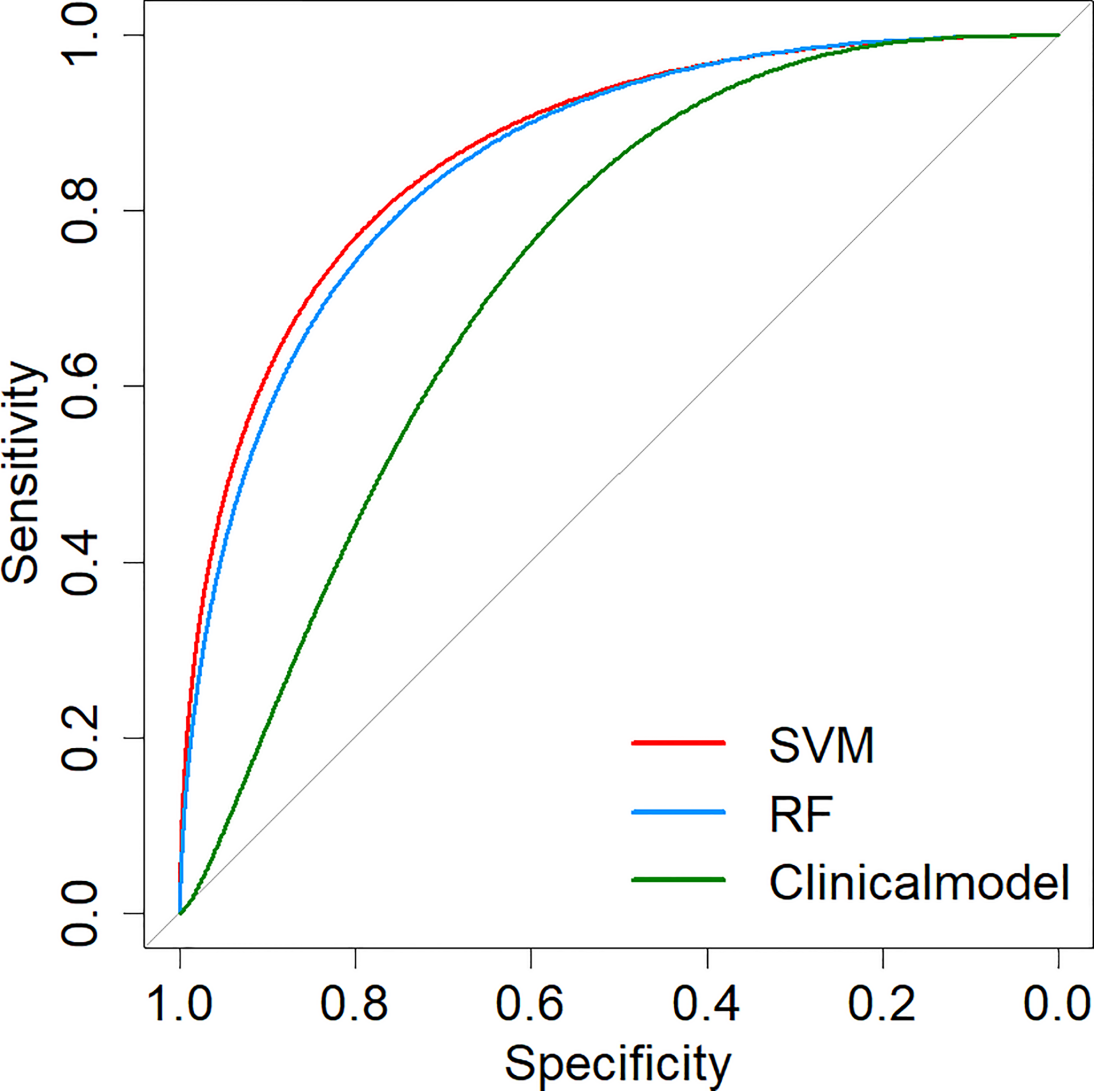
ROC curves of prediction models. SVM, Support Vector Machine; RF, Random Forest; ROC, Receiver operating characteristic.

Two machine learning models and a clinical model were validated in the validation cohort. The SVM (AUC: 0.876, ACC: 0.800, SEN: 0.667, SPE: 0.941) and RF (AUC: 0.863, ACC: 0.800, SEN: 0.667, SPE: 0.941) models performed well and correctly distinguished between LUAD and LUSC. The Clinical model had moderate predictive performance (AUC:0.712, ACC: 0.686, SEN: 0.882, SPE: 0.500). The predicted performance of the model is shown in **Table 2**. The receiver operating characteristic (ROC) curves of the models are shown in **Figure 6**. The DeLong test was used to compare the performance of the three models. There was no significant difference between the SVM model and the RF model (P=0.825), but the SVM model was significantly better than the Clinical model (P=0.037). The difference between the RF model and Clinical model was not statistically significant (P=0.144). The model comparison results are shown in **Table 3**.

**Table 2:** Comprehensive performance of prediction models for predicting NSCLC subtypes in the validation group.

Model	AUC (95%CI)	ACC (95%CI)	SEN (95%CI)	SPE (95%CI)
SVM	0.876 (0.761-0.990)	0.800 (0.791-0.809)	0.667 (0.449-0.884)	0.941 (0.829-1.053)
RF	0.863 (0.742-0.983)	0.800 (0.791-0.809)	0.667 (0.449-0.884)	0.941 (0.829-1.053)
Clinical model	0.712 (0.547-0.878)	0.686 (0.674-0.698)	0.882 (0.729-1.036)	0.500 (0.269-0.731)

The area under the receiver operating characteristic curve, AUC; Sensitivity, SEN; Specificity, SPE; Accuracy, ACC; Support Vector Machine, SVM; Random Forest, RF.

**Table 3:** DeLong test within different prediction models.

Model-1	Model-2	P value
SVM	RF	0.825
SVM	Clinical model	0.037
RF	Clinical model	0.144

Support Vector Machine, SVM; Random Forest, RF.

## Discussion

Convenient and low-risk methods of distinguishing between LUAD and LUSC have significant clinical significance, as the two differ in terms of their biological characteristics, clinical characteristics, and prognosis. In this study, we have completed two main works. First, we constructed 10 machine learning classifiers and determined that SVM (AUC: 0.876, ACC: 0.800, SEN: 0.667, SPE: 0.941) and RF (AUC: 0.863, ACC: 0.800, SEN: 0.667, SPE: 0.941) models were more suitable classifiers for the classification task of NSCLC. Secondly, we tried to combine clinical factors-laboratory metrics-radiomic features to construct prediction models and compared them with the Clinical model. The results showed that the input of multiple factors could help the classifier better characterize the tumor to some extent. Importantly, we proposed a noninvasive method for differentiating NSCLC subtypes that can assist in the clinical identification of different pathological subtypes of NSCLC, particularly in cases where patients are unsuitable for biopsy or where biopsy fails.

Recently, radiomics has been combined with machine learning to distinguish NSCLC subtypes. Alvarez-Jimenez et al. constructed an SVM with a linear kernel based on the radiomic features of CT images to classify LUAD and LUSC. Their method had an AUC of 0.72 ± 0.01 (95% CI: 0.65–0.77) and accuracy of 0.69 ± 0.01 (95% CI: 0.61–0.74) (19). Zhu et al. found that a radiomic signature computed using five CT radiomic features correctly distinguished between the LUAD and LUSC, with AUCs of 0.905 and 0.893 in the training and validation cohorts, respectively (20). Different machine learning models may perform differently for the task of classification of NSCLC, so the choice of the classifier is helpful to explore the optimal solution to this problem, which has been preliminarily explored in a previous study (12). Considering that most previous studies only explored one model, we further determined the best algorithm for this binary classification task in this experiment by comparing 10 common machine learning models to determine the best classifier (SVM and RF) for the classification of NSCLC. After optimizing the parameters of the models, we further verify the validity of the model in the validation cohort. Although the SVM model was significantly superior to the Clinical model, the difference between the RF model and the Clinical model was not statistically significant. However, it is hopeful that the RF model proposed by us could improve the performance compared with the Clinical model. The reason for no significant difference is that limited by small data sets; it is not possible to determine statistically whether the machine learning model is superior to the Clinical model (21). For both machine learning models and Clinical models, the calculated AUC indicated that the machine learning classifiers had high diagnostic accuracy.

Most traditional feature selection algorithms follow a minimal optimization method that relies on a small subset of features and produces minimal errors in selection classification. We use the Boruta algorithm to filter features. Boruta follows all the relevant feature selection methods, and it can capture all the features related to the result variable. In this study, the Boruta algorithm returned 3 PET radiomic features and 6 CT radiomic features in the dominant feature subset. Since all tumor scales (macro, physiological, micro, and genetic) are heterogeneous, structural and metabolic heterogeneity (22, 23). This study extracted the radiomic features from PET and CT images. As a multimodal image, PET/CT explores the internal tumor heterogeneity in terms of both anatomy and function. Although the signal-to-noise ratio and resolution of PET images are poor, radiomic features extracted based on PET images can reveal tumor metabolic heterogeneity, which is an informative supplement to CT radiomic features that can only respond to anatomical heterogeneity. This was demonstrated by Koyasu et al., who obtained PET/CT images of a group of lung cancer patients (156 LUAD and 32 LUSC) from a public database (13). Through Bayesian optimization, a gradient tree boosting model was constructed with optimal radiomic features consisting of metabolic indices in PET, histograms of CT, histograms of PET, and local binary patterns of CT. The accuracy of their model reached 0.830 when distinguishing LUAD from LUSC (13). Han et al. built an LDA model using 50 PET/CT radiomic features. They achieved optimal predictive performance (AUC of 0.863 and accuracy of 0.794), further confirming the value of the PET/CT radiomic features combined with machine learning to distinguish LUAD from LUSC (12).

In addition, the Boruta algorithm and univariate analysis showed that clinical factors and laboratory indicators were also helpful in differentiating NSCLC subtypes. This is because men with a smoking history are at higher risk of LUSC (24, 25). Serum tumor markers levels are susceptible in the diagnosis of non-small cell lung cancer (26). SCCA in lung SCC patients is significantly higher than that in lung ADC patients, while CEA is at a low level (10, 27). Recent reports have further demonstrated the positive role of clinical factors and laboratory indicators (10, 11). In a retrospective analysis involving 315 NSCLC patients, the authors used a least absolute shrinkage and selection operator regression model with two clinical factors, two tumor markers, seven PET radiomic, and three CT radiomic features to predict NSCLC subtypes (10). They confirmed that the combined multi-factor constructed model performed better than using them alone (10). While the linear model is simple, more complex models may provide more accurate tools for clinical practice. Therefore, Hyun et al. constructed 5 machine learning models with these two clinical factors (gender and age) and 13 PET radiomic features (11). Their results showed that the LR model had the best predictive performance, with an AUC value of 0.859 and an ACC of 0.769 (11). Unfortunately, this study only considered clinical factors and PET radiomic factors.

Inspired by previous studies, we, for the first time attempted to combine clinical factors, laboratory indicators, PET and CT radiomic features to construct prediction models, and identified the most suitable prediction model for the subtypes of NSCLC. In other words, we have a better description of the tumor, and this study is a further supplement to the previous research results. We note that Han et al. explored the value of the VGG16 deep learning model in differentiating NSCLC subtypes based on PET/CT images, and the predictive performance of the VGG16 deep learning model was superior to that of traditional machine learning models (12). This is an exciting result, but comparing with traditional machine learning models, deep learning models require a larger sample size for training (12). In addition, the training cost of the deep learning model also needs to be solved. Although the results from different study cohorts cannot be directly compared, the SVM and RF models proposed by our study are accurate and convenient. SVM model has the advantages of being suitable for small sample machine learning data and strong generalization ability, and this algorithm is very convenient for binary classification tasks (28). RF model is an integration algorithm based on the DT model. One of the significant advantages of the RF model is that it can maintain model accuracy even if there is missing data (29, 30), which is very suitable for clinical application scenarios because it is not always possible to ensure that all clinical information of patients is complete in clinical practice. As a result, RF and SVM models constructed in this study are significant for the clinical identification of NSCLC subtypes.

In addition, the replication and validity of the radiomic feature extraction process are essential for translating potential applications into clinical practice (31, 32). Semi-automatic segmentation seems more conducive to the realization of this goal. Although manual segmentation was used in this study, we assessed the segmentation reproducibility inter-and intra-class and excluded one feature with poor reproducibility. Strict reproducibility detection increased the generalizability of the results of this experiment.

Our experimental results are encouraging, but several limitations in our study should be noted. First, additional testing should be performed for the generalization of the model. Because the sample size was small and came from a single medical institution, the model may not be robust. Second, although as many characteristic parameters as possible were included in our study, a proportion of patients lacked clinical factors and laboratory indicators. As such, other characteristics might further enhance the performance of the model. Third, the ratio of LUAD and LUSC in the sample data was different from real cases. To ensure the performance of radiomic features, primary lesions in some LUAD patients were excluded because they showed weak [^18^F]F-FDG uptake or small tumor volume. This led to the proximity of LUAD to LUSC patients in the experiment. Thus, further evaluation is needed to determine whether other samples affect our model.

## Conclusion

The proposed machine learning models constructed with clinical factors, laboratory indicators, and [^18^F]F-FDG PET/CT radiomic features can assist with the clinical identification of LUAD and LUSC. The model is convenient, noninvasive, and accurate, and it is especially suitable in cases where patients are unsuitable for biopsy or where biopsy fails.

## Data Availability Statement

The original contributions presented in the study are included in the article/**Supplementary Material**. Further inquiries can be directed to the corresponding author.

## Ethics Statement

The studies involving human participants were reviewed and approved by The Ethics Review Board of The First Affiliated Hospital of Harbin Medical University. Written informed consent for participation was not required for this study in accordance with the national legislation and the institutional requirements.

## Author Contributions

HZ and YS provided the overall design of the experiments and contributed equally to the experiments. PX, YJ, LZ, WH, and LT were responsible for collecting clinical and imaging data. MW and ZL completed tumor segmentation. HZ and YS performed the radiomics analysis and model building. HZ, YS, and PF wrote and edited the manuscript. PF supervised the study. All authors contributed to the article and approved the submitted version.

## Funding

This work was supported by funds from Scientific Research and Innovation Fund of the First Affiliated Hospital of Harbin Medical University (No. 2021M32).

## Conflict of Interest

The authors declare that the research was conducted in the absence of any commercial or financial relationships that could be construed as a potential conflict of interest.

## Publisher’s Note

All claims expressed in this article are solely those of the authors and do not necessarily represent those of their affiliated organizations, or those of the publisher, the editors and the reviewers. Any product that may be evaluated in this article, or claim that may be made by its manufacturer, is not guaranteed or endorsed by the publisher.
